# The antidepressant and anxiolytic effects of cannabinoids in chronic unpredictable stress: a preclinical systematic review and meta-analysis

**DOI:** 10.1038/s41398-022-01967-1

**Published:** 2022-05-31

**Authors:** Noa Reuveni, Cole A. Carlson, Sarah Schwartz, Diana Meter, Tyson S. Barrett, Sara M. Freeman

**Affiliations:** 1 Department of Psychology, Utah State University, Logan, UT USA; 2 Department of Biology, Utah State University, Logan, UT USA; 3 Department of Human Development and Family Studies, Utah State University, Logan, UT USA

**Keywords:** Molecular neuroscience, Depression

## Abstract

Neuroscience research presents contradictory evidence in support of both the protective and destructive effects of cannabinoids in depression. Therefore, this systematic review and meta-analysis summarizes the existing preclinical literature on the effects of cannabinoid administration in the chronic unpredictable stress model of depression in order to evaluate the effects of cannabinoids and identify gaps in the literature. After protocol registration (PROSPERO #CRD42020219986), we systematically searched Scopus, Embase, Psychology & Behavioral Sciences Collection, APA PsychINFO, PubMed, CINAHL Complete, and ProQuest Dissertations & Theses Global from the earliest record of the databases, February 1964, to November 2020 for articles that met inclusion criteria (e.g., rodent subjects and administration of a cannabinoid. A total of 26 articles were included representing a sample size estimate of 1132 rodents with the majority of articles administering daily intraperitoneal injections during chronic unpredictable stress. These articles were evaluated using a modified SYRCLE’s risk-of-bias tool. For each continuous behavioral measure, the standardized mean difference was calculated between cannabinoid and vehicle groups in rodents subjected to chronic unpredictable stress. The effects of cannabinoids on depressive-like behavior was evaluated using a multilevel mixed-effects model with effect size weights nested within control groups. Cannabinoid administration moderately improved the pooled negative effects of chronic unpredictable stress on anhedonia, learned helplessness, novelty suppressed feeding, time in the anxiogenic context, and entries into the anxiogenic context. Although the interpretations are limited, these findings suggest that with further investigation, cannabinoids may be a viable long-term treatment for stress-related psychopathologies such as depression.

## Introduction

According to the World Health Organization, “depression is a leading cause of disability worldwide” [1]. Depression can lead to suicide, which is the “second leading cause of death in 15–29 years olds” [1]. Furthermore, the global impact of depression and related disorders continues to increase [1]. Current antidepressants have therapeutic delays, low efficacy, and increased risk of suicide, indicating a need for alternative pharmacotherapies for depression [2, 3].

The chronic unpredictable stress paradigm (CUS) models depression in laboratory rodents [4]. To effectively model depressive phenotypes, the CUS paradigm exposes rodents to a variety of frequently occurring, intermittent stressors for several weeks [4]. CUS induces passive-coping behaviors such as learned helplessness (e.g., increased immobility in the Porsolt forced swim test) and anhedonia (i.e., reduced interest in sweetened water) that reflects the diagnostic criteria of major depressive disorder (MDD) [4, 5]. CUS-induced anhedonia can be reversed with chronic (but not acute) antidepressant treatment, demonstrating the paradigm’s high predictive validity [6–8]. Furthermore, stimulants, which can result in false positives for acute stress models of depression, do not reverse depressive symptoms in CUS [8]. This paradigm’s high face validity is demonstrated with chronic elevations of the stress hormone corticosterone (CORT) in rodents, reflecting elevations of the stress hormone cortisol in MDD patients [4, 7, 9]. Furthermore, MDD patients demonstrate significant reductions in hippocampal volume and brain-derived neurotrophic factor (BDNF) expression in comparison to healthy controls [10]. Comparatively, mice expressing the BDNF Val66Met polymorphism demonstrate reductions in hippocampal volume and anxious phenotypes [10]. For a review on the role of BDNF and neurogenesis in depression in both the clinical and preclinical literature, please see ref. [10].

CUS dysregulates the brain and body’s stress response. In the brain, the prefrontal cortex (PFC) coordinates psychological reactivity to stress [11]. Generally, the neuroendocrinological pathology of depression involves stress circuits, such as the ventral subiculum of the hippocampus and basolateral amygdala (BLA), relaying respective inhibitory and excitatory signals to the paraventricular nucleus of the hypothalamus (PVN) which initiates the hypothalamic pituitary adrenal (HPA) axis stress response [11]. HPA axis activation is initiated by the secretion of corticotropin-releasing hormone (CRH) by the PVN, followed by the secretion of adrenocorticotropic hormone (ACTH) by the anterior pituitary [11]. This cascade leads to the secretion of glucocorticoids (GC) by the adrenal cortex, such as CORT in rodents, which then initiates negative feedback of the HPA axis [11]. High circulating levels of GCs in instances of chronic stress can decrease glucocorticoid receptor (GR) expression and increase dendritic atrophy in the hippocampus, thereby contributing to the reduction of hippocampal volume observed in MDD [4, 10, 11].

In the pursuit of alternative pharmacotherapies for depression, preclinical evidence suggests that pharmacological enhancement of the endogenous cannabinoid system (ECS) promotes antidepressant and anxiolytic effects [2, 8, 12–22]. The fundamental elements of the ECS consist of the cannabinoid one receptor (CB1R), the cannabinoid two receptor (CB2R), the endogenous cannabinoid (eCB) arachidonoyl-ethanolamide (i.e., anandamide) (AEA), the eCB 2-arachidonoyl glycerol (2-AG), and the enzymes involved in eCB synthesis and catabolism [23]. CB1R is the most abundant G protein coupled receptor in the central nervous system, while the CB2R is typically located on immune cells [23–25]. AEA is considered as a partial agonist at cannabinoid (CB) receptors similarly to tetrahydrocannabinol (THC), whereas 2-AG has greater efficacy at CB receptors than AEA [24, 25]. Cannabidiol (CBD) has been considered an antagonist at CB receptors, but recent research suggests that CBD could be a allosteric modulator at CB receptors [26]. Finally, *β*-Caryophyllene (BCP) is a selective CB2R agonist [18].

Several synthetic cannabinoids (CBs) have been developed to target various aspects of the ECS. WIN55,212–2 is a CB1R and CB2R agonist, and HU-210, is a potent non-selective CB1R agonist [12, 27]. Arachidonyl-2-chloroethylamide (ACEA) is administered for selective CB1R agonism [28]. Common antagonists include Rimonabant (RIM) and AM251, which are CB1R antagonists with inverse agonist effects when chronically administered [3, 13, 21, 29, 30]. For the CB2R, JWH133 and JWH015 are selective CB2R agonists, while AM630, is a CB2R antagonist with inverse agonist effects when chronically administered [19, 21, 28, 31]. Overall, the main catabolic enzyme for AEA is fatty acid amide hydrolase (FAAH), while the main catabolic enzyme for 2-AG is monoacylglycerol lipase (MAGL), and the compounds URB597 and JZL184 are enzyme inhibitors for FAAH and MAGL, respectively [14, 15, 17, 23, 32–34].

Both the preclinical and clinical literature indicates a relationship between CBs and depression. Cross-sectional studies suggest behavioral correlations between cannabis use and depression [35]. Twin and family studies also suggest common genetic factors between cannabis use and depression [35]. Although it is possible that depressive symptoms increase cannabis use rather than the inverse association, it does not appear that chronic cannabis use has a long-term positive effect on depressive symptoms [35]. According to National Surveys on Drug Use and Health from 2008–2019, increases in suicide ideation, plan, and attempt are associated with varying degrees of cannabis use [36]. Early-onset chronic cannabis use is also correlated with dysregulated cortisol rhythms, suggesting dysregulated HPA axis activity [25]. These clinical data highlight a need to further investigate the relationship between CBs and depression. Mounting preclinical evidence supports the hypotheses by Hill and colleagues that AEA levels in the BLA gatekeep HPA axis activation and that GC release activates GR which mobilize the synthesis of eCBs to facilitate a rapid mechanism for negative feedback of the HPA axis [25]. For a review on eCB signaling and the HPA axis please see ref. [25]. This evidence suggests that eCB signaling facilitates an adaptive stress response, demonstrated with rapid and effective activation of the HPA axis followed by a timely and complete termination of the HPA axis [4, 25].

The current study aims to identify the conditions in which exogenous administration of CBs promotes stress vulnerability and the conditions in which exogenous administration of CBs promotes stress resilience. Meta-analyses pose a unique benefit in organizing existing evidence and identifying gaps in the literature. The goals of this study are to facilitate the development of CB-based pharmacotherapies for stress-related psychopathologies by identifying gaps in the literature, to discourage unnecessary preclinical research designs, and to encourage preclinical research designs with greater external validity [37, 38].

## Methods

### Overview

This research adheres to the CAMRADES guidelines for conducting systematic reviews and meta-analyses of animal studies with a preregistered research protocol in the Systematic Review Protocol for Animal Intervention Studies (SYRCLE) format [38–40]. This protocol is publicly available in the Systematic Review Facility (SyRF) protocol database, PROSPERO protocol database (registration number: CRD42020219986), and on the Open Science Framework (https://osf.io/csgmf/) in order to facilitate visibility and transparency [40]. Protocol deviations can be viewed in Supplementary Table 1. All articles were screened, coded, and evaluated in a standardized manner by two independent reviewers (NR and CC). Disagreements between reviewers were resolved by discussion, and the final decision was made by a third independent reviewer (SF).

### Criteria and search strategy

**Fig. 1:**
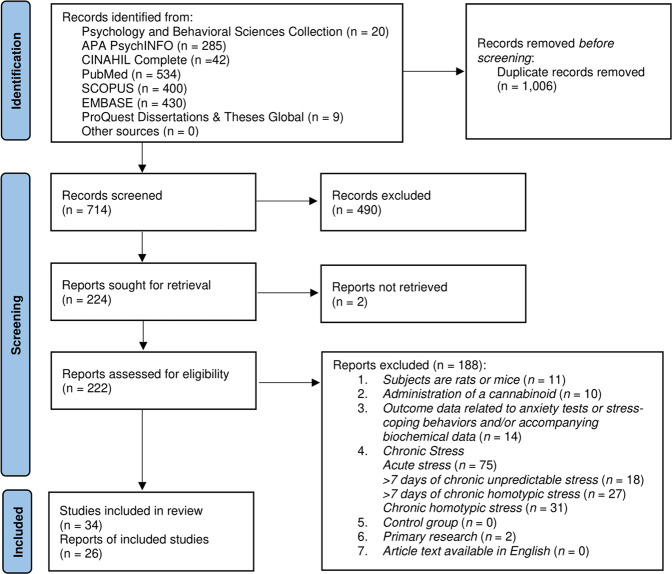
PRISMA flowchart [67]. Flowchart of the literature search and selection process.

Inclusion criteria and search strategy are presented in Fig. 1. Please see supplemental information for additional details regarding inclusion criteria and search strategy. Briefly, potential articles were identified by searching Scopus, Embase, Psychology & Behavioral Sciences Collection, APA PsychINFO, PubMed, CINAHL Complete, and ProQuest Dissertations & Theses Global from the earliest record of the databases, February 1964, to November 2020. Search terms included rat OR mouse AND cannabinoids AND chronic stress. Articles were deemed eligible for the meta-analysis if they met the following inclusion criteria: (1) study subjects were either rats or mice [37, 41, 42]; (2) subjects received administration of a CB; (3) Outcome data related to anxiety tests (e.g., open field test) or stress-coping behaviors (e.g., self-grooming) and/or accompanying biochemical data (e.g., related to the endogenous cannabinoid system or hypothalamic pituitary adrenal axis stress response); (4) subjects were exposed to variable heterotypic stressors for more than seven days; (5) there was a study-matched control group; (6) the article was the primary source of the research (i.e., not a review); (7) the article was available in English. For the third inclusion criterion pertaining to outcome measures, any outcome deemed to measure stress or anxiety was included and no specific tests were required for inclusion. Neuroendocrinological measures of stress were considered to be scientific techniques used in neuroscience and endocrinology (e.g., CORT measures) in articles that met the remaining criteria.

### Data collection

All coding was completed using a custom, online REDCap form that was developed based on CAMRADES guidelines [38, 43]. For a complete list of extracted data fields, the REDCap codebook file is attached to the preregistered protocol. Please see supplemental information for additional details regarding data collection.

### Quality assessment

The quality of included articles was assessed by evaluating the potential for bias using SYRCLE’s risk-of-bias tool, which was developed to establish consistency in preclinical article evaluation [39]. SYRCLE’s risk-of-bias tool was adapted to include additional evaluation measures. Please see supplemental information for additional details regarding quality assessment.

### Statistical analysis

Although all behavioral and neuroendocrinological measures were included in our dataset, meta-analyses were only conducted for the most commonly reported behavioral measures (i.e., anhedonia, learned helplessness, novelty suppressed feeding, time in the anxiogenic context, and entries into the anxiogenic context). In the interest of achieving sufficient statistical power for each specific behavioral measure. Based on previous meta-analysis protocols, we decided in our preregistered protocol that a meta-analysis will be carried out if there are more than three studies with the same outcome. Four studies had the distance traveled outcome for exploration anxiety tests. Therefore, a meta-analysis was conducted for the distance traveled outcome but was not considered to be part of the most commonly reported behavioral measures since it was less powered in comparison to the other outcomes. Although it is possible to run a meta-analysis with four studies, the interpretation is relatively limited. Furthermore, there were a variety of measures collected for exploration anxiety tests, and the two most common measures were the time spent in the anxiogenic context and entries into the anxiogenic context. Time and entries were analyzed separately due to inconsistent results for each measure in the same exploration anxiety test, suggesting that time and entries in the anxiogenic context capture different aspects of anxiety (e.g., locomotion) [21].

**Fig. 2:**
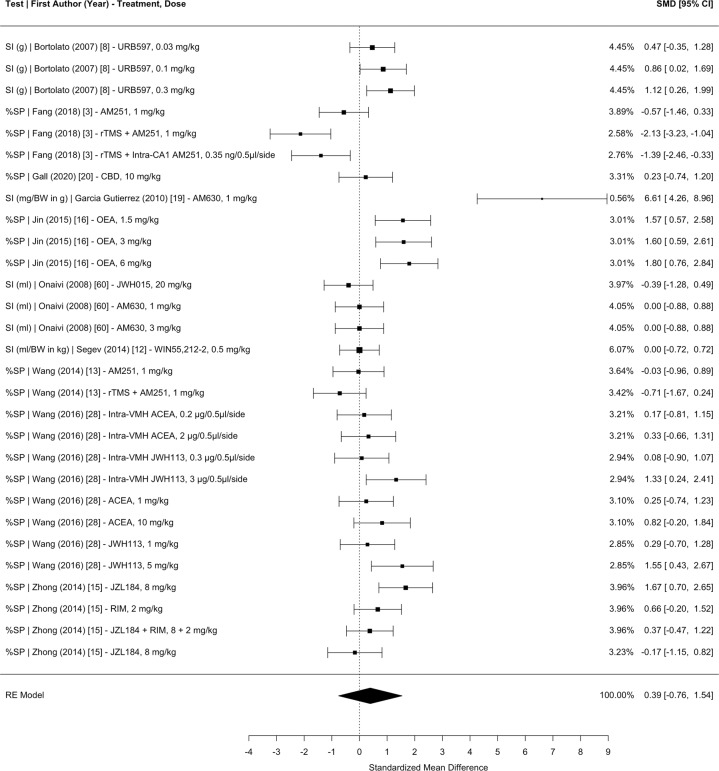
Meta-analysis of studies investigating the effect of CB administration on anhedonia (i.e., sucrose intake or preference) in CUS. SI sucrose intake, %SP percent sucrose preference, BW body weight, SMD standardized mean difference, CI confidence interval, rTMS repetitive transcranial magnetic simulation, CBD cannabidiol, OEA oleoylethanolamide, VMH ventromedial hypothalamus, CA1 hippocampal cornu ammonis 1, ACEA arachidonyl-2-chloroethylamide.

**Fig. 3:**
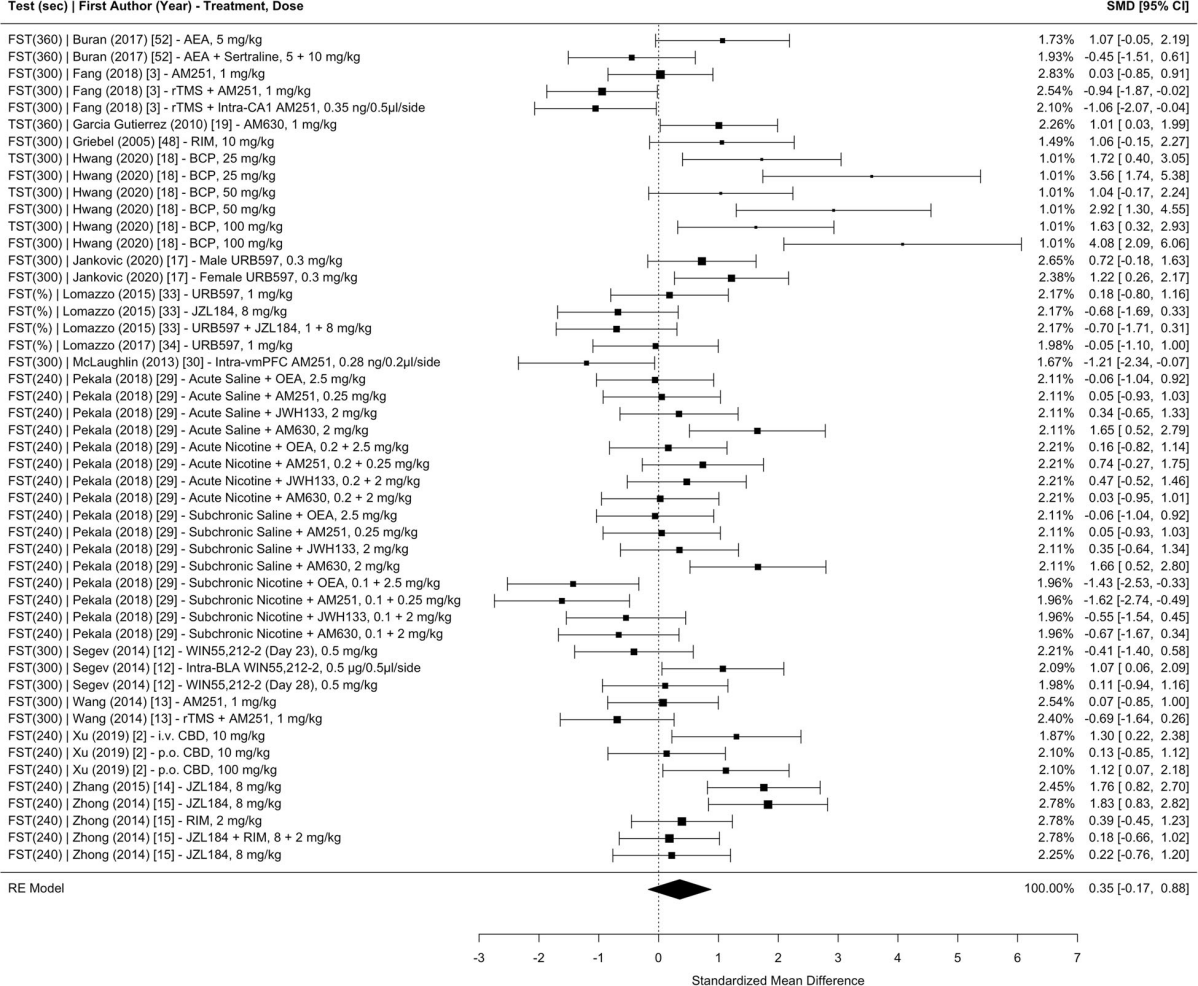
Meta-analysis of studies investigating the effect of CB administration on learned helplessness (i.e., immobility time or percent immobility for the forced swim test or tail suspension test) in CUS. Sec total time of the test in seconds, FST forced swim test, TST tail suspension test, SMD standardized mean difference, CI confidence interval, AEA anandamide, rTMS repetitive transcranial magnetic simulation, RIM rimonabant, BCP *β*-caryophyllene, vmPFC ventromedial prefrontal cortex, OEA oleoylethanolamide, BLA basolateral amygdala, CBD cannabidiol.

**Fig. 4:**
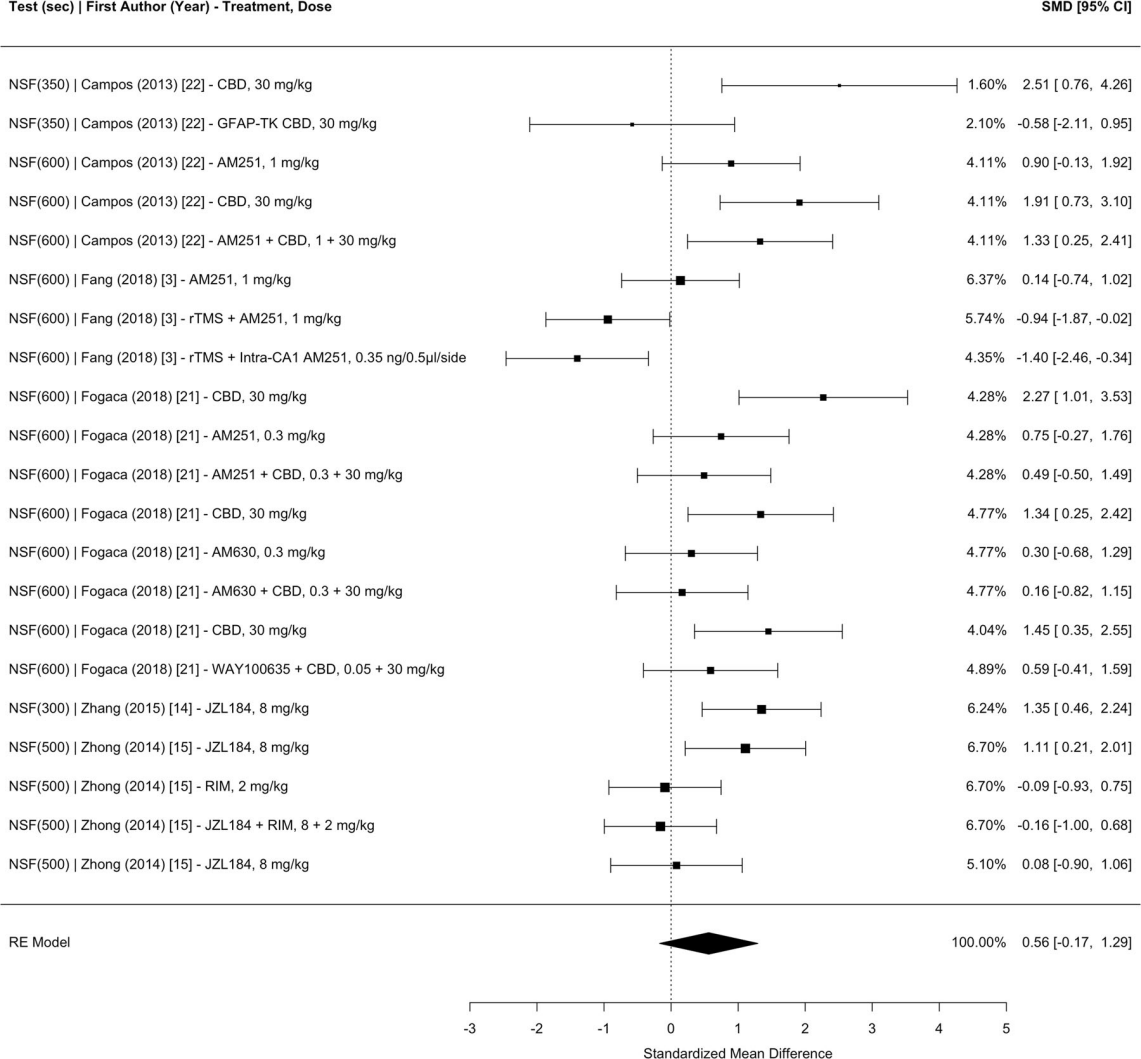
Meta-analysis of studies investigating the effect of CB administration on anxiety in the novelty suppressed feeding test (i.e., latency to consume food in a novel environment) in CUS. SMD standardized mean difference, CI confidence interval, CBD cannabidiol, GFAP-TK glial fibrillary acidic protein thymidine kinase transgenic mice, rTMS repetitive transcranial magnetic simulation, CA1 hippocampal cornu ammonis 1, RIM rimonabant.

**Fig. 5:**
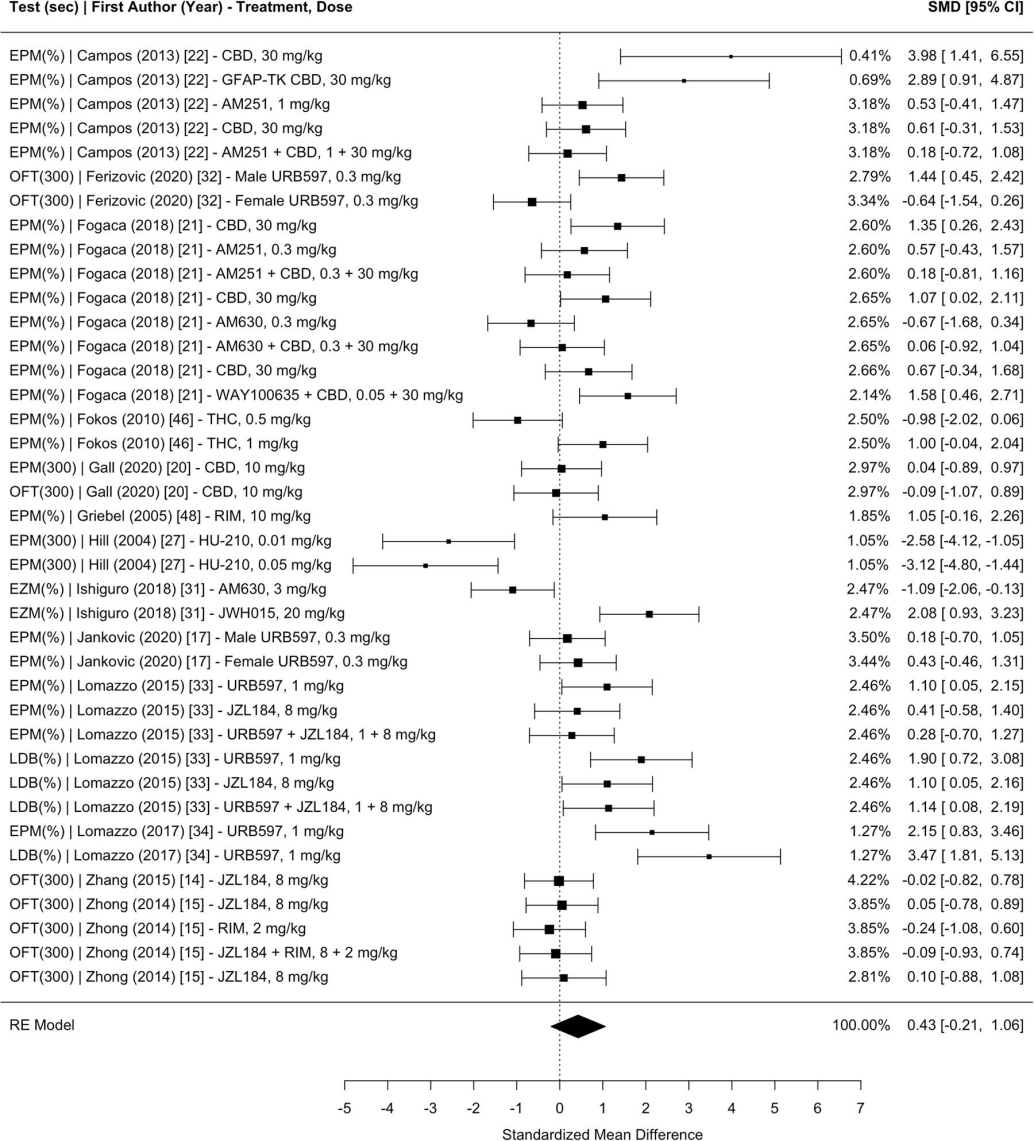
Meta-analysis of studies investigating the effect of CB administration on exploration anxiety (i.e., % or time spent in the anxiogenic context) in CUS. Sec total time of the test in seconds, EPM elevated plus-maze, OFT open field test, EZM elevated zero maze, LDB light dark box test, SMD standardized mean difference, CI confidence interval, CBD cannabidiol, GFAP-TK glial fibrillary acidic protein thymidine kinase transgenic mice, THC tetrahydrocannabinol, RIM rimonabant.

**Fig. 6:**
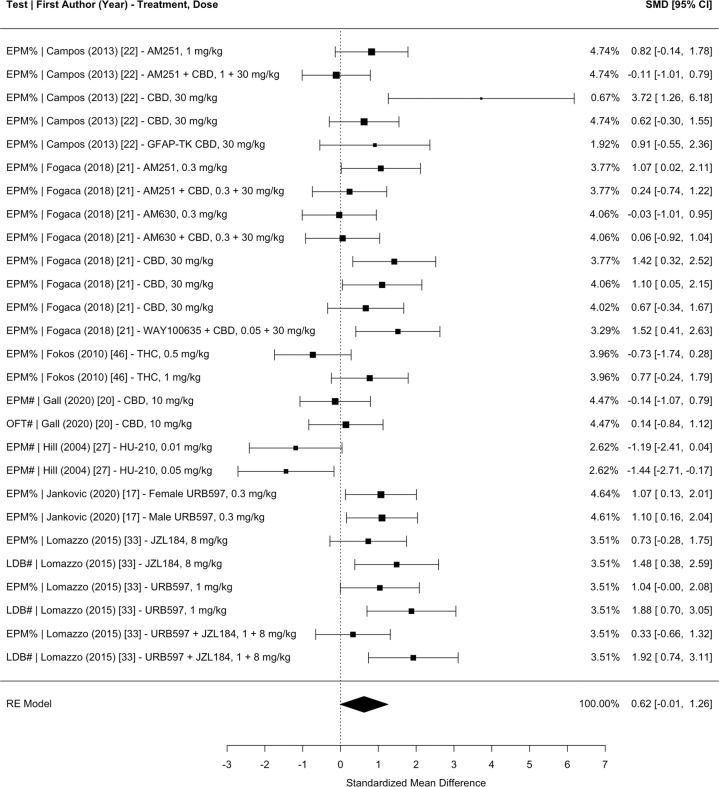
Meta-analysis of studies investigating the effect of CB administration on exploration anxiety (i.e., % or # of entries into the anxiogenic context) in CUS. EPM elevated plus-maze, OFT open field test, LDB light dark box test, SMD standardized mean difference, CI confidence interval, CBD cannabidiol, GFAP-TK glial fibrillary acidic protein thymidine kinase transgenic mice, THC tetrahydrocannabinol.

A random-effects model was used to account for the variance due to methodological heterogeneity [38]. For meta-analyses, each effect size is considered to be completely independent. However, due to the nature of preclinical research (e.g., having multiple experimental groups for each control group), the effect sizes tend to be correlated with each other, resulting in dependencies [38]. In the present study, five levels of dependencies were identified: laboratories, articles, study groups (e.g., research design), measurement type (e.g., using the same group of rodents for multiple tests), and control groups (Supplementary Fig. 1). Instead of assuming independent effect sizes, a multilevel model was used to model effect size dependencies between articles, study groups, and measurement type [38]. Please see supplemental information for a full description of dependencies. To address control group level dependency, if there were multiple different CB-treated CUS groups compared to a single vehicle-treated CUS group, the effect size was calculated for each group, and a nested weight based on precision which is regarded as the inverse of variance was created so that every effect size compared to the same control group shared the same weight. This nested control group weight allowed for separate effect size measurements while addressing dependency between measurements with the same control group. In addition to this pooled model, separate subgroup meta-analyses were also conducted for each test measurement as an additional method to address measurement type-level dependency.

For continuous measures of stress-coping behaviors (e.g., immobility time in seconds in a forced swim test), the standardized mean difference (SMD) effect size between vehicle-treated CUS and CB-treated CUS groups was calculated. The SMD, also known as Hedge’s G, is similar to Cohen’s D because they both utilize a pooled standard deviation [44]. Hedge’s G is usually preferred in meta-analyses, because this effect size corrects for biases in small sample sizes [38, 44]. A restricted maximum likelihood estimation method was used in the models to avoid underestimation of the variance or bias in the estimation of variance [38].

Moderator analyses can identify sources of between-study heterogeneity in effect sizes and characteristics which influence the overall effect of CBs on stress-coping behaviors in CUS. Identification of significant moderators can provide guidance for the experimental design of future preclinical studies [38]. Categorical moderators of this meta-analysis were species (rats vs. mice), method of administration (systemic vs. site-specific), the timing of administration relative to CUS (during vs. after vs. both), the overall effect on the ECS (enhancement vs. inhibition vs. neutral) and co-administration (yes vs. no). Continuous moderators were the dose of the CB, number of administrations, and days of stress. Species were considered as a moderator according to a previous meta-analysis [45]. The method of administration, the overall effect on the ECS, co-administration, dose of CB, and a number of administrations were all included as moderators in consideration of the biphasic effect of CBs on stress where systemic administration of low doses of CB1 agonists are anxiolytic and high doses of CB1 agonists are anxiogenic [25, 46–48]. Timing of administration relative to stress was also considered to investigate differences in the effects of CBs on stress management and recovery from stress. We decided to code for the overall effect on the ECS in order to be able to include co-treatment administration and in the interest of maintaining sufficient power for this moderator analysis. Further categorizing CB administration by the specific CB or by its pharmacological targets would have likely prevented certain moderators from being meaningfully interpreted due to insufficient power. In one article, days of stress were given in a range, so the lowest value was used for a conservative estimate [19]. For the two articles that administered CBs every two days, the number of administrations was counted by assuming that the first administration began on the first day [14, 15]. There was no missing moderator data. However, it is important to note that there were not enough effect sizes to be able to conduct two proposed moderator analyses for the effects of the model (control vs. disease) and sex (males vs. females) with three effect sizes for disease models and four effect sizes for females. With such few effect sizes to analyze, it would be difficult to interpretate moderators that are underpowered.

Potential publication bias from funnel plot asymmetry was assessed using Egger regression [49]. Heterogeneity was evaluated using the *I*^2^ statistic with *I*^2^ of more than 25%, 50%, and 75% selected to reflect low, moderate, and high heterogeneity, respectively [50].

## Results

### Article selection and characteristics

Some studies appeared to meet all inclusion criteria but were excluded upon closer investigation during the review. For example, one article conducted chronic mild stress and administered the CB WIN55,212–2 [51]. However, the group that received WIN55,212–2 only underwent acute elevated platform stress and was therefore excluded because the subjects that were administered a CB were not exposed to variable heterotypic stressors for more than seven days [51].

Out of the 26 articles that met all inclusion criteria, 21 articles contained studies in which CBs were administered through intraperitoneal injections, 19 articles contained studies in which CBs were administered during CUS, and 15 articles contained studies in which CBs were administered daily (Table 1). The proportion of types of stressors administered can be viewed in Supplementary Table 2. Pharmacological co-treatments were all able to be controlled for with a corresponding study-matched control group [21, 29, 38, 52]. In one study, repetitive transcranial magnetic simulation (rTMS) treatment was administered to both the vehicle-treated CUS and CB-treated CUS groups [3]. Therefore, the effect sizes from this article were kept in the meta-analysis and addressed in the co-treatment moderator analysis as a co-treatment [3]. Finally, one article conducted an additional intervention of a middle cerebral artery occlusion surgery that was performed on all CUS subjects [28]. This article was kept in the meta-analysis, and this additional intervention was addressed by adding a “high” evaluation score to be factored into the bias score for the sensitivity analysis (https://osf.io/csgmf/) [28, 45].

**Table 1:** Preclinical article characteristics.

Author, year	Subjects	Stress schedule	Stress types	Treatment(s)	Administration schedule
Bortolato et al., 2007 [8]	200 g Male Wistar Rats	70 days (2–3/day)	9	URB597 0.03, 0.1, and 0.3 mg/kg (i.p.)	Daily—last 35 days of CUS (35 total)
Buran, Etem, Tektemur, & Elyas, 2017 [52]	40 g Male BALB/c Mice (90 days old)	49 days (2–3/day)	11	AEA 5 mg/kg (s.c.) and Sertraline 10 mg/kg (i.p.)	After CUS (3 total)
Campos et al., 2013 [22]	Male GFAP-TK and C57BL/6 J Mice (90 days old)	14 days (1/day)	6	CBD 30 mg/kg (i.p.) and AM251 1 mg/kg (i.p.)	Daily—concurrent with CUS (14 total)
Fang & Wang, 2018 [3]	Male Sprague Dawley Rats (21 days old)	21 days (2–3/day)	8	rTMS and AM251 1 mg/kg (i.p.) or 0.35 ng/0.5 μl/side (intra-CA1)	Daily—7 days after CUS (7 total)
Ferizovic, Spasojevic, Stefanovic, Jankovic, & Dronjak, 2020 [32]	250–300 g Male and Female Wistar Rats (77 days old)	42 days (2/day)	13	URB597 0.3 mg/kg (i.p.)	Twice daily—last 14 days of CUS (28 total)
Fogaça, Campos, Coelho, Duman, & Guimarães, 2018 [21]	20–26 g Male C57BL/6 J Mice (56–63 days old)	14 days (1/day)	8	CBD 30 mg/kg (i.p.), AM251 0.3 mg/kg (i.p.), AM630 0.3 mg/kg (i.p.), and WAY100635 0.05 mg/kg (i.p.)	Daily—concurrent with CUS (14 total)
Fokos & Panagis (2010) [46]	270–320 g Male Sprague Dawley Rats	10 days (2/day)	7	THC 0.5 and 1 mg/kg (i.p.)	After CUS (1 total)
Gall et al., 2020 [20]	355–419 g Adult Male Wistar Rats	28 (2/day)	11	CBD 10 mg/kg (i.p.)	Daily—concurrent with CUS and 4 days after (32 total)
García-Gutiérrez, Pérez-Ortiz, Gutiérrez-Adán, & Manzanares, 2010 [19]	25–35 g Male Swiss ICR Mice (60–90 days old)	49–56 days (1–3/day)	7	AM630 1 mg/kg (i.p.)	Twice daily—last 28 days of CUS (56 total)
Griebel, Stemmelin, & Scatton, 2005 [48]	20–27 g Male BALB/c Mice	49 days (1–3/day)	4	Rimonabant 10 mg/kg (p.o.)	Daily—last 35 days of CUS (35 total)
Hill & Gorzalka, 2004 [27]	300 g Male Long Evans Rats (70 days old)	21 (3/day)	6	HU-210 10 and 50 μg/kg (i.p.)	After CUS (1 total)
Hwang et al., 2020 [18]	167–183 g Male Sprague Dawley Rats (49 days old)	28 days (1–2/day)	4	*β*-Caryophyllene 25, 50, and 100 mg/kg (i.p.)	Daily—concurrent with CUS (28 total)
Ishiguro et al., 2018 [31]	20–25 g Male C57BL/6 J Mice (56–70 days old)	14 (2/day)	6	AM630 3 mg/kg (i.p.) and JWH015 20 mg/kg (i.p.)	Daily—concurrent with CUS (14 total)
Jankovic, Spasojevic, Ferizovic, Stefanovic, & Dronjak, 2020 [17]	250–300 g Male and Female Wistar Rats (77 days old)	42 days (2/day)	13	URB597 0.3 mg/kg (i.p.)	Twice daily—last 14 days of CUS (28 total)
Jin, Yu, Tian, Zhang, & Quan, 2015 [16]	18–22 g Adult Male Kunming Mice	28 days (2/day)	14	Oleoylethanolamide 1.5, 3, 6 mg/kg (p.o.)	Daily—last 21 days of stress (21 total)
Lomazzo et al., 2015 [33]	Male C57BL/6 J Mice (42 days old)	73 days (2–3/day)	17	URB597 1 mg/kg (i.p.) and JZL184 8 mg/kg (i.p.)	Daily—last 38 days of stress (38 total)
Lomazzo, Köing, Abassi, Jelinek, & Lutz, 2017 [34]	Male C57BL/6 J Mice (42 days old)	73 days (2–3/day)	17	URB597 1 mg/kg (i.p.)	Daily—last 38 days of stress (38 total)
McLaughlin et al., 2013 [30]	300 g Male Sprague Dawley Rats (70 days old)	21 days (2–3/day)	8	AM251 0.28 ng/0.2 μl/side (intra-vmPFC)	After CUS (1 total)
Onaivi et al., 2008 [60]	Male and Female BALB/c Mice	28 days	6	JWH015 20 mg/kg (i.p.) and AM630 1 and 3 mg/kg (i.p.)	Daily—concurrent with CUS (28 total)
Pekala, Michalak, Kruk-Slomka, Budzynska, & Biala, 2018 [29]	20–25 g Male Swiss Mice (60 days old)	27 days (1/day)	7	Nicotine 0.1 and 0.2 mg/kg (s.c.), Oleoylethanolamide 2.5 mg/kg (i.p.), AM251 0.25 mg/kg (i.p.), JWH133 2 mg/kg (i.p.), and AM630 2 mg/kg (i.p.)	After CUS (1 total)
Segev, Rubin, Abush, Richter-Levin, & Akirav, 2014 [12]	Male Sprague Dawley Rats (45 days old)	21 days (1–2/day)	8	WIN55,212–2 0.5 mg/kg (i.p.) and 5 μg/0.5 μl/side (intra-BLA)	Daily—last 3 days of CUS (i.p. 3 total); After CUS (intra-BLA 1 total)
Wang et al., 2014 [13]	180–230 g Male Sprague Dawley Adult Rats	28 days (1–4/day)	14	rTMS and AM251 1 mg/kg (i.p.)	Daily—7 days after CUS (7 total)
Wang et al., 2016 [28]	250–270 g Male Sprague Dawley Adult Rats	18 days	9	Middle cerebral artery occlusion surgery, ACEA 1 and 10 (i.p.) or 0.2 and 2 μg/0.5 μl/side (intra-VMH), JWH133 1 and 5 mg/kg (i.p.) or 0.3 and 3 μg/0.5 μl/side (intra-VMH)	Daily—first 7 days of CUS (i.p. 7 total); After CUS (intra-VMH 1 total)
Xu et al., 2015 [2]	32–38 g Male ICR Mice (42 days old)	35 days	4	CBD 10 (i.v.), 10, and 100 (p.o.)	Weekly—last 28 days of CUS (4 total)
Zhang et al., 2015 [14]	Male C57BL/6 J Mice (56–70 days old)	35 days (1–2/day)	11	JZL184 8 mg/kg (i.p.)	Every 2 days—last 14 days of stress and 7 days after (11 total)
Zhong et al., 2014 [15]	17–25 g Male C57BL/6 J Mice (56–70 days old)	35 days (2/day)	11	JZL184 8 mg/kg (i.p.) and Rimonabant 2 mg/kg (i.p.)	Every 2 days—21 days of CUS and 7 days later (14 total); Every 2 days—last day of CUS and 7 days later (4 total)

*CUS* Chronic unpredictable stress, *GFAP-TK* glial fibrillary acidic protein thymidine kinase transgenic mice, *AEA* anandamide, *CBD* cannabidiol, *rTMS* repetitive transcranial magnetic simulation, *THC* tetrahydrocannabinol, *i.p.* intraperitoneal, *s.c.* subcutaneous, *p.o.* oral, *CA1* hippocampal cornu ammonis 1, *vmPFC* ventromedial prefrontal cortex, *ACEA* arachidonyl-2-chloroethylamide, *i.v.* intravenous, *BLA* basolateral amygdala, *VMH* ventromedial hypothalamus.

### Qualitative analysis

All CB-related measurements between the vehicle-treated CUS and CB-treated CUS groups were included in Supplementary Table 3. Overall, the inconsistent findings summarized in this table emphasize the need to further investigate the effects of CUS on AEA levels in the hippocampus [8, 33, 53, 54]. When summarizing stress-related measurements, only the most common measurements between the vehicle-treated CUS and CB-treated CUS groups were included in the interest of creating a cohesive overview (Supplementary Table 4). Briefly, this table highlights the CBs which alleviate CUS-induced decreases in BDNF expression and the CBs which alleviate CUS-induced decreases in neurogenesis [16]. A complete table of the qualitative data is available on the Open Science Framework (https://osf.io/csgmf/).

### Quantitative analysis

The main mixed-effects model, which nested the effect sizes within measurement type, study groups, and articles (with effect size weights nested within control groups), revealed a significant pooled SMD of 0.4456 (95% CI 0.0498–0.8415, *p* = 0.0274), indicating that CB administration moderately improves the overall negative effects of CUS on anhedonia, learned helplessness, novelty suppressed feeding, time in the anxiogenic context, and entries into the anxiogenic context in comparison to placebo. Separate meta-analyses nesting the effect sizes within study groups and articles with effect size weights nested within control groups were conducted for each test measurement: (1) anhedonia 0.3919 (95% CI −0.7580 to 1.5417, *p* = 0.5042; Fig. 2); (2) learned helplessness 0.3520 (95% CI −0.1725 to 0.8765, *p* = 0.1884; Fig. 3); (3) novelty suppressed feeding 0.5614 (95% CI −0.1716 to 1.2943, *p* = 0.1333; Fig. 4); (4) time spent in the anxiogenic context 0.4273 (95% CI −0.2056 to 1.0602, *p* = 0.1857; Fig. 5); (5) entries into the anxiogenic context 0.6231 (95% CI −0.0095 to 1.2558, *p* = 0.0535; Fig. 6). It is possible that these effects were unable to reach significance because they have less statistical power than the main model. These separate analyses suggest that it might be worthwhile to continue to investigate the effect of CBs on entries into the anxiogenic context in CUS. In the separate meta-analysis for learned helplessness (i.e., immobility time), an exploratory moderator analysis of the effect of the studies’ test time revealed that test time did not significantly affect the SMD *β* = 0.2244 (95% CI −0.0376 to 0.4865, *p* = 0.0932), indicating that the immobility time measurement was not significantly affected by the test time.

Moderator analyses were conducted using a multilevel model, which nested effect sizes within study groups and articles and with effect size weights nested within control groups. All moderator analyses included two moderators with measurement type as the second moderator. For CB treatments that did not include an additional treatment (i.e., not a co-treatment), there was a significant SMD of 0.7919 (95% CI 0.1387–1.4450, *p* = 0.0175) while CB treatments that included an additional treatment revealed a SMD of 0.0544 (95% CI −0.7331 to 0.8420, *p* = 0.8923). This co-treatment moderator analysis is likely a better representation of the efficacy of CBs on stress-coping behaviors in CUS, since a sizeable amount of co-treatment effect sizes combined CB agonists with CB antagonists. In support of this interpretation, a moderator analysis revealed that for CBs that enhance the ECS, there was a significant SMD of 0.7809 (95% CI 0.1281–1.4337, *p* = 0.0190). Meanwhile, CBs that inhibit the ECS revealed a SMD of 0.0792 (95% CI −0.6421 to 0.8005, *p* = 0.8296) and CBs that have an overall neutral effect on the ECS revealed a SMD of 0.2267 (95% CI −0.7127 to 1.1661, *p* = 0.6362). These data support the continuation of research on the effects of CBs that enhance the ECS on stress-coping behaviors in CUS.

Some categorical moderators were limited by the number of effect sizes in their category. For example, a moderator analysis revealed that for CB administration during CUS, there was a significant SMD of 0.8043 (95% CI 0.3022–1.3065, *p* = 0.0017). These data suggest that CBs are more efficacious at regulating the HPA axis during CUS rather than after CUS. For the timing of CB administration relative to CUS, the “both” and “after” moderators should be interpreted with caution. The “both” moderator should be interpreted with caution due to statistical power, with 97 effect sizes for the “during” moderator, 49 effect sizes for the “after” moderator, and only 19 effect sizes for the “both” moderator. The “after” moderator should be interpreted with caution because a proportion of the effect sizes administering CBs after CUS also administered CBs once intracranially. In another instance, the model was not significantly moderated by the method of administration. For CBs administered systemically, there was SMD of 0.6231 (95% CI −0.0453 to 1.2916, *p* = 0.0677) and for CBs administered in a site-specific manner there was a SMD of −0.0280 (95% CI −1.1401 to 1.0841, *p* = 0.9607). However, the method of administration moderator analysis should be interpreted with caution, as there were only nine effect sizes with site-specific intracranial administration.

Continuous moderators revealed that the effects of CBs on stress-coping behaviors in CUS is significantly moderated by the number of CB administrations and dose of the CB. Every additional administration significantly increased the SMD by a value of *β* = 0.0262 (95% CI 0.0026–0.0499, *p* = 0.0295). Hillard and colleagues hypothesize that the effects of CBs on the HPA axis depend on the eCB tone with a high eCB tone facilitating effective HPA axis termination [25]. Therefore, it is possible that chronic CB treatment artificially maintains a high eCB tone. Furthermore, every additional mg/kg significantly increased the SMD by a value of *β* = 0.0209 (95% CI 0.0078–0.0339, *p* = 0.0018). It is important to take into consideration that the dose moderator was conducted on a dataset that removed site-specific and co-treatment effect sizes. The effect of dose might reflect the development of tolerance to CBs and the need to maintain this high eCB tone.

Species differences were observed in this dataset and the moderator analysis revealed that for mice there was a significant SMD of 0.7772 (95% CI 0.0511–1.5032, *p* = 0.0359) and for rats there was a SMD of 0.2856 (95% CI −0.4262–0.9974, *p* = 0.4316). Although the differences between rats and mice exposed to CUS have not been formally investigated, it appears that mice might have higher stress reactivity than rats [55]. If this observation is true, then it is possible that CBs exhibit greater efficacy on mice because mice have a greater reaction to CUS. Regardless, further investigation is needed on the differences between species on the effects of CBs on stress-coping behaviors in CUS.

Surprisingly the model was not significantly moderated by the number of days of stress *β* = 0.0096 (95% CI −0.0122 to 0.0315, *p* = 0.3878). These data suggest that the effects of CBs on stress-coping behaviors in CUS is not significantly affected by the severity of the CUS paradigm. Therefore, it might be more impactful for future studies to focus less on the severity of the CUS paradigm and more on conducting CUS with under-studied groups, such as female rodents or disease models (e.g., for depression and anxiety) to investigate the effects of CBs on stress-coping behaviors in CUS in these populations. The need to investigate this gap in the literature is further emphasized by the lack of a sufficient number of effect sizes that were needed to conduct the proposed moderator analyses on the effects of the model (control vs. disease) and sex (males vs. females).

### Bias assessment

**Fig. 7:**
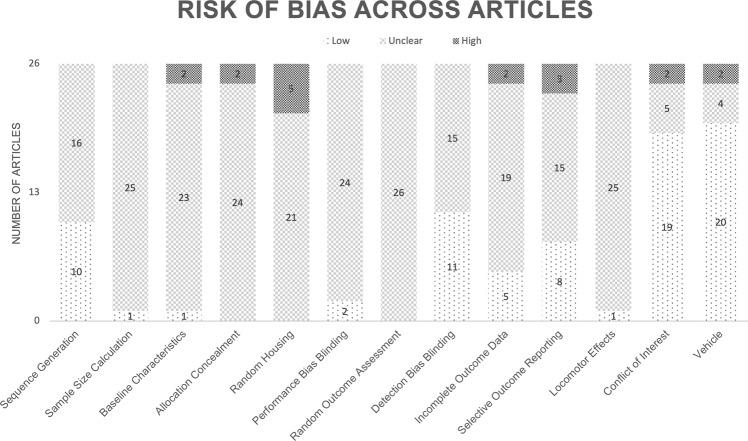
Evaluation of included articles. Aside from the conflict of interest statement and vehicle measures, an unclear risk of bias is the most common evaluation score across all measures.

To successfully replicate and further research, it is important to effectively communicate the methods of a study. As observed in Fig. 7, the most common evaluation score across all measures aside from the conflict of interest and vehicle measures is “unclear”, which indicates a need for more explicit reporting of measures taken to reduce the risk of bias in order to mitigate the possibility that the effects are overestimated due to bias [45]. When evaluating baseline characteristics, all articles reported sex and usually either the weight or age of the subjects. In neuroscience research, all characteristics are important when considering measures such as the effect of a treatment or the effect on the subject’s brain. When conducting research with rodents, it is not always possible to control for litter effects initially due to housing constraints. However, it is possible to statistically control for the variance shared by littermates, which can reduce the likelihood of committing an error [56]. When evaluating allocation concealment, most articles were “unclear” about how the CUS schedule was generated. Two articles explicitly stated that the same weekly schedule was repeated, which can negatively impact the efficacy of the CUS paradigm since the subjects are more likely to habituate to a predictable weekly schedule in comparison to a completely randomized schedule [4].

When evaluating random housing, several articles received “high” scores by stating that the experimental group was housed in a different room than the control group. Housing different groups in different rooms can result in disproportionate exposure to noise and differences in colony maintenance by animal technicians, thereby influencing stress-coping behaviors. Furthermore, environmental conditions such as illumination and temperature can differ between cages on the top and bottom shelf [39]. Since differences in illumination and temperature can also influence stress-coping behaviors, environmental conditions can be controlled by counterbalancing shelf locations between groups [39, 57]. In other instances, the housing conditions were different between the control and experimental groups (i.e., individually or group-housed). With social stressors such as isolation and overcrowding, it is important that the housing conditions are comparable between control and experimental groups since the housing conditions can also influence stress-coping behaviors [56].

When evaluating incomplete outcome data, although two articles received “high” scores, five of the more recent articles received “low” scores for reporting the initial size of their groups, the group sizes for analysis, and communicating sources of attrition or exclusions in their article. In alignment with encouraging this trend towards explicit communication of group sizes, it would also be impactful to communicate sample size calculations. Low statistical power, which is commonly observed in preclinical research, has tremendous consequences on the reliability and reproducibility of results due to overestimated effect sizes [58].

When evaluating selective outcome reporting, although three articles received “high” scores, it is important to recognize the eight articles with “low” scores explicitly stated when data were not shown in their article. However, considering that there is additional space to provide complete datasets with non-significant results it is important to encourage the publication of negative findings in supplementary material or preprint servers. Making all possible information available and accessible upon publication entirely circumvents the need to directly request additional information from researchers who might not always be able to easily locate their old records. Therefore, encouraging researchers to provide all of their data in the non-graphical form and all analysis calculations has immense potential to facilitate the reproducibility of work and to improve the quality of meta-analytic work, which will ultimately reduce unnecessary animal use [41].

Compared to other bias evaluations, there is a greater proportion of conflicts of interest statements, reporting of blinding, and randomization of groups (i.e., sequence generation). These trends of increased reporting in preclinical research provide hope for continued improvement in reporting the measures taken to reduce the risk of bias [59]. It is also interesting to note that despite a subset of articles that monitored body weight and sucrose preference throughout stress to validate the CUS paradigm, only one article investigated the locomotor effects of the drugs administered [60]. When investigating exploration anxiety, it would be impactful to screen for the locomotor effects of the drugs administered or to provide a baseline within-subject measure of locomotion to control for locomotor effects of the drug. For example, a different exploratory anxiety pretest can be implemented to collect comparable outcome measures while preventing habituation to the test. A within-subject baseline locomotion measurement in exploration anxiety tests would be an effective standard control measurement similarly to how the latency to consume food in the home cage is a standard control measurement for the novelty suppressed feeding test. This within-subject measure that is taken in addition to measuring the latency to consume food in a novel environment is used to control for possible anorexigenic or orexigenic effects of drugs. Knowing that cannabis facilitates sleepiness and stimulates appetite emphasizes the importance of these controls [25].

In line with controlling for the effects of a drug, it is also important to control for the stress-inducing effects of injections and any effects of the solvent that is administered to the experimental group by administering the same exact solution without the drug (i.e., a vehicle) to the control group. Therefore, vehicle administration was added as an evaluation criterion.

A separate sensitivity analysis was conducted by generating a bias score which can be viewed in the article-level moderators file in the Open Science Framework (https://osf.io/csgmf/). This bias score was calculated for each article by adding one point for every “low” evaluation score and subtracting one point for every “high” evaluation score, then adding two points to each article so that the lowest bias score was zero. In this sensitivity analysis, two articles were removed for having the lowest bias scores, revealing a significant SMD of 0.4547 (95% CI 0.0388–0.8705, *p* = 0.0321) which is not substantially different than the pooled SMD of 0.4456 (95% CI 0.0498–0.8415, *p* = 0.0274).

### Publication bias and heterogeneity

The main mixed-effects meta-analysis model is a multilevel model (i.e., effect sizes nested within measurement type, study groups, and articles) and utilizes effect size weights nested within control groups to address multiple levels of dependencies. Currently, there is no single method to evaluate publication bias while addressing sample dependency [61]. Ignoring dependency and assuming that this meta-analysis is a univariate model rather than a multilevel mixed-effects model can inflate the type I error rate [61]. Therefore, two analyses for publication bias were conducted. The first analysis conducted an Eggar regression on a complete funnel plot with the nested weights. Whereas the second analysis conducted an Eggar regression on a subset funnel plot with the nested weights, representing the single largest effect size for each of the 26 included articles (assuming that articles are published based on their largest effect size). An Egger regression on the funnel plot of the full dataset (Supplementary Fig. 2a) revealed the significant presence of asymmetry (t = −5.005, df = 163, *p* < 0.001). In comparison to the Egger regression on the funnel plot of the full dataset, an Egger regression on the funnel plot of the subset data (Supplementary Fig. 2b) approached the significant presence of asymmetry (t = −2.053, df = 24, *p* = 0.0511). Conflicting findings from these two methods of analysis have been previously reported [61]. Since the Egger regression on the funnel plot of the subset data approached the significant presence of asymmetry, these Eggar regressions suggest that publication bias is present in the dataset. An asymmetrical funnel plot indicates a lack of non-significant and opposing findings, therefore, suggesting selective publishing of significant and positive effects [45].

In a multilevel mixed-effects model nesting the effect sizes within study groups and articles but not within measurement type, there was a moderate amount of heterogeneity across studies (*I*^2^ = 69.5218%), indicating inconsistent results. As expected based on methodological heterogeneity, there was more heterogeneity between studies (*I*^2^ = 46.4898%) than within studies (*I*^2^ = 23.0319%). Separate analyses by test measurement identified anhedonia as the most heterogenous measurement (*I*^2^ = 91.2125%) with a much greater proportion of heterogeneity between studies (*I*^2^ = 86.1742%) than within studies (*I*^2^ = 5.0382%). Entries in the anxiety context was the least heterogenous measurement (*I*^2^ = 64.5820%) with more heterogeneity between studies (*I*^2^ = 51.2359%) than within studies (*I*^2^ = 13.3462%).

The most effective moderator in reducing heterogeneity was the time the drug was administered relative to CUS (*I*^2^ = 56.6541%). None of the heterogeneity was between studies (*I*^2^ = 0%), and all of the heterogeneity was within studies (*I*^2^ = 56.6541%), indicating that all the variances of effect sizes by this moderator were due to sampling error or chance [38]. Although it is tempting to speculate that this within-study heterogeneity might be due to differences from significant moderators that were not identified in this meta-analysis, these data suggest a need to control for this moderator in future investigations on the effect of CBs administered during CUS. To better elucidate this source of heterogeneity, it would be helpful to conduct a study that investigates this moderator further by comparing CB administration that is concurrent with the stressors to the administration that occurs in between the stressors.

## Discussion

This systematic review and meta-analysis provides an overview of the existing preclinical literature on the effects of CB administration on stress-coping behaviors in CUS. A total of 26 articles were included in this study which revealed that CB administration moderately improved the overall negative effects of CUS on anhedonia, learned helplessness, novelty suppressed feeding, time in the anxiogenic context, and entries into the anxiogenic context. Moderator analyses revealed that this effect significantly increased with increasing doses and number of CB administrations, an effect that should be further investigated in the context of the biphasic effect of CBs. Furthermore, this effect was significantly greater (1) in mice than in rats, (2) with a CB that enhances the ECS, and (3) when the CB is administered during CUS. These findings highlight gaps in the literature and emphasize a need to investigate these effects in females and disease models. In conclusion, further investigation is needed to determine if CBs are a viable long-term treatment for stress-related psychopathologies such as depression. Although these results are valuable in guiding the design of future preclinical research, the translational implications of these results should be interpreted with caution due to limitations in reporting measures taken to reduce the risk of bias, heterogeneity, small sample sizes, publication bias, and effect size dependencies.

Moderator analyses revealed that the protective effects of CBs were significantly greater in mice compared to rats, which supports previous findings that reported differences between rodent species on the effects of CBs in anxiety measurements (i.e., the proportion of time and entries into open arms) in the elevated plus-maze [57]. In a study from Haller and colleagues, WIN55,212–2 (at a dose of 3 mg/kg in mice and 1 mg/kg in rats) was anxiolytic in mice and anxiogenic in rats [57]. In this same study, AM251 (at a dose of 3 mg/kg in mice and 5 mg/kg in rats) was anxiogenic in mice and had no effect on rats [57]. Furthermore, differences between mouse strains in stress reactivity have been reported [4]. Generally, BALB/c mice are considered as stress vulnerable, C57BL/6 J mice are considered as stress resilient, and CD1 mice demonstrate greater variability in stress reactivity [4]. In line with these findings, future research on the effects of CBs on rodent models of depression and anxiety in comparison to “control” models are needed. There is also a need for research on sex differences in the effects of CB on stress-coping behaviors in CUS. Research on sex differences in stress-coping behaviors in CUS can be immensely impactful when considering the increased prevalence of stress-related psychopathologies in females compared to males [4]. Unfortunately, there were not enough effect sizes to be able to conduct the proposed moderator analyses for the effects of the model (control vs. disease) and sex (males vs. females), which again highlights the need for the inclusion of these groups in future studies.

The findings of this meta-analysis should be interpreted with caution, as evaluation of these articles revealed that the most common risk of bias score across all measures aside from the conflict of interest statement and vehicle administration was “unclear” [39]. Based on these bias evaluations, it is important to not disregard the potential for bias in the included articles. Reporting the measures taken to reduce the risk of bias and being transparent about the measures that were not taken is an effective approach to reduce unnecessary animal use and facilitate the reproducibility of research results. When journals require reporting of measures taken to reduce the risk of bias (e.g., disclosing sample size calculations), there appears to be a substantial improvement in reporting the measures taken to reduce the risk of bias.

A second limitation in meta-analysis research pertains to methodological heterogeneity. Heterogeneity was quantified from *I*^2^ in order to appropriately analyze and best interpret the predictive validity of the meta-analysis results [50, 62]. Although a random-effects model was selected to best demonstrate the distribution of the studies estimated effects, results revealed moderate heterogeneity across studies indicating inconsistent results, which negatively impacts the predictive validity of findings [45, 63]. The most effective moderator in reducing heterogeneity was the time the drug was administered relative to CUS, which suggests that CBs have different effects depending on when they are administered in relation to stressors.

A third limitation in meta-analysis research are small sample sizes, which reduces the reliability and therefore the validity of the studies’ outcomes [58, 64]. Although there was a total of approximately 1132 rodents in this meta-analysis, experimental groups ranged from three to fifteen rodents per group. Experiments with small sample sizes are not always published which can lead to publication bias [45, 58]. Therefore, the fourth limitation in this meta-analysis is publication bias which was demonstrated by funnel plot asymmetry suggesting that non-significant and opposing findings might not be published [45]. In consideration of these limitations, the sources of heterogeneity and evaluation of biases still provide valuable guidance in improving the external validity of future studies. A significant pooled SMD despite moderate heterogeneity might suggest that the main effect of our meta-analysis is quite robust and that CBs may be efficacious at improving the overall negative effects of CUS in a variety of contexts, therefore supporting the external validity of CB efficacy [62].

In the interest of statistical power, the definition of the CUS paradigm was selected to be as inclusive as possible, and the most common stress-coping behavioral tests were subjected to the meta-analysis. Although there were 165 effect sizes included in this meta-analysis, the statistical power of this considerably large number of effect sizes was negatively impacted by dependencies. The dependencies were addressed with a multilevel mixed-effects model and with effect size weights nested within control groups. However, attempts to elucidate sources of heterogeneity were less successful. It is also possible that there are sources of methodological heterogeneity that were not reported in the articles, thereby contributing to the limitations of meta-analysis research [38].

The results of this current study summarize the existing preclinical literature from mice and rats on the effects of CBs on stress-coping behaviors in CUS. Despite revealing a significant pooled SMD in the model, the focus of this meta-analysis was to guide future preclinical study designs rather than evaluate the overall efficacy of CBs. These data highlight gaps in the literature by suggesting the sample characteristics for which such pharmacotherapies should be preclinically tested and designed for. Furthermore, these data can help guide future preclinical research away from unnecessary replication of study designs and from studies that appear less efficacious [38]. Identifying the study designs which appear less efficacious will refocus research efforts toward preclinical studies that are more translationally impactful. Overall, these data provide an initial framework for the future directions of preclinical research investigating CB-based pharmacotherapies for stress-related psychopathologies. In alignment with the guidance this meta-analysis provides, the bias evaluation in this meta-analysis echoes the importance of reporting research methods. A clear presentation of methods may reduce the risk of bias in preclinical research in order to facilitate reproducible research and improve the external validity of preclinical research.

## Supplementary information

Supplement

Supplementary Table 1

Supplementary Figure 1

Supplementary Table 2

Supplementary Figure 2

Supplementary Table 3

Supplementary Table 4

## Data Availability

The excel files containing the full dataset with study level moderators and article-level moderators as well as the R markdown code file containing all statistical analyses are available on the Open Science Framework (https://osf.io/csgmf/). The meta-analysis was performed using the *metafor* package in R (v4.0.2) software [65, 66].
