# Supplementary material for: The antidepressant and anxiolytic effects of cannabinoids in chronic unpredictable stress: a preclinical systematic review and meta-analysis
Source: Transl Psychiatry. 2022 May 31;12:217. doi: 10.1038/s41398-022-01967-1 (PMC9156762; doi:10.1038/s41398-022-01967-1)
Supplement: Supplementary file 1 — Supplement [file 41398_2022_1967_MOESM1_ESM.docx]

**Supplementary Information**

Methods

Figures S1-S2

Tables S1-S5

1. **Methods**

**Criteria**

Articles were deemed eligible for the meta-analysis if they met the following inclusion criteria: 1) study subjects were either rats or mice [1-3]; 2) subjects received administration of a CB; 3) outcome measures included behavioral and/or neuroendocrinological stress; 4) subjects were exposed to variable heterotypic stressors for more than seven days; 5) there was a study matched control group; 6) the article was the primary source of the research (i.e. not a review); 7) the article was available in English. Although this last criteria poses as an inherent bias against non-English articles, there appears to be minimal benefit in the translation of non-English articles [4]. Conference abstracts were omitted due to limited information. In the second stage of screening, the reason for removal of the article was documented and reported (Figure 1) in order facilitate transparency of the screening process [1]. The inclusion criterion of “variable heterotypic stressors for more than seven days” was applied in the second stage of screening in order to prioritize severe and previously validated CUS paradigms. Protocol deviations such as these can be viewed in Supplementary Table 5.

**Search Strategy**

Potential articles were identified by searching Scopus, Embase, Psychology & Behavioral Sciences Collection, APA PsychINFO, PubMed, CINAHL Complete, and ProQuest Dissertations & Theses Global from February 1964 to November 2020. The libraries were imported into EndNote X9 and Mendeley for automatic then manual de-duplication which was replicated at least three separate times to ensure that no reference was deleted by accident. Search terms included rat OR mouse AND cannabinoids AND chronic stress. The complete search strategy including the exact search syntax used for each database is located at the end of the preregistered protocol. All articles were screened in a standardized manner by two independent reviewers (N.R. and C.C). First, the title and abstract for each reference was reviewed to identify potential articles before reviewing the full-text to determine article eligibility. Disagreements between reviewers were resolved by discussion and the final decision was made by a third independent reviewer (S.F.). In included articles, citations were tracked both forwards and backwards to search for additional articles. Both forms of citation tracking did not result in additional included articles, indicating that there was no need for further citation tracking of excluded reviews. Authors of included studies, relevant investigators at the National Institute of Health, and at the Hebrew University Multidisciplinary Center on Cannabinoid Research were contacted for grey literature. In total, 53 authors were individually contacted for grey literature in efforts to include all relevant data. Thirteen of those 53 emails received a response, and two of those responses contained article suggestions that were later excluded. Finally, authors of included studies were contacted and asked to provide additional study details and data when needed. Twenty-nine additional emails were sent out to request additional information. Out of those 29 additional emails, two responded saying they did not have the requested information, one agreed to send their data but was unable to find it, and one researcher responded with a raw dataset that corresponded to an included article. This raw dataset was used for a validation analysis by comparing the raw dataset to data extracted by the two independent reviewers using the WebPlotDigitizer software [5]. After the validation analysis, the raw dataset was used for the meta-analysis instead of the reviewer-extracted data. An additional exploratory search was conducted based on article recommendations provided by the Mendeley reference manager software, which yielded 36 additional articles that were later excluded.

**Data Collection**

All coding was completed using an online custom REDCap form that was developed based on CAMRADES guidelines [6, 7]. For a complete list of extracted data fields, the REDCap codebook file is attached to the preregistered protocol. Data presented in graphical format was extracted using WebPlotDigitizer graph digitization software [5]. A validation analysis compared the raw dataset provided from the researcher that responded, to the data that was extracted by the two independent reviewers using the WebPlotDigitizer software [5]. The means of the extracted dataset were within 3% of the raw dataset, while the standard errors of the means of the extracted dataset were within 13% of the raw dataset. If the final group sizes were given in a range, then the lowest number within that range was collected for a conservative estimate. Twenty-one articles used the standard error of the mean for the error bars in their figures, and one article used standard deviation. Therefore, the four articles which did not specify their error bars were assumed to be standard error of the mean. Unfortunately, this limitation in reporting variance has been previously observed in preclinical research [6]. Finally, for studies with repeated measures, data was only collected for the last measurement. All forms of data collection were conducted by two independent reviewers (N.R. and C.C). Disagreements between reviewers on article characteristics and neuroendocrinological data were resolved by discussion, and the final decision was made by a third independent reviewer (S.F.). For the behavioral data, measurements that were not within half a unit of each other or within one unit of each other for the novelty suppressed feeding test were recalculated by both independent reviewers until they were within said range, then averaged. The standardized mean difference (SMD) calculations between both independent reviewers were also confirmed to be within 0.05 of each other.

**Quality Assessment**

Potential bias was evaluated using a modified version of SYRCLE’s risk-of-bias tool [8]. SYRCLES’s risk-of-bias tool was adapted to include four additional evaluation measures: control for the locomotor effects of CBs, sample size calculations, conflict of interest statements, and proper vehicle administration [9]. Risk-of-bias evaluations were conducted by two independent reviewers (N.R. and C.C). Disagreements between reviewers on risk-of-bias evaluations were resolved by discussion, and the final decision was made by a third independent reviewer (S.F.).

**Statistical Analysis Description of Dependencies**

In the present study, five levels of dependencies were identified: laboratories, articles, study groups, measurement type, and control groups (Supplementary Figure 1). At the laboratory level, research is usually published from a similar group of investigators sharing the same equipment. The laboratory level was the only level of dependency that was not able to be addressed by the models since the remaining levels were prioritized as greater sources of dependencies. Laboratory level dependency can overlap with article level dependency. Examples of laboratory and article level dependencies include shared subject pools, protocols, and analysis methods [10]. At the study level, a single article can consist of multiple correlated studies sharing similar research designs [6]. At the measurement type level, the same group of subjects can be used for multiple tests and multiple different measurements can be collected from the same test [6]. At the control group level, the same measurements from multiple experimental groups are compared to the same control group [6].

**2 References Cited**

1. Leenaars, M., Hooijmans, C.R., van Veggel, N., ter Riet, G., Leeflang, M., Hooft, L., et al., *A step-by-step guide to systematically identify all relevant animal studies.* Lab Anim, 2012. **46**(1): p. 24-31.

2. Hooijmans, C.R., Tillema, A., Leenaars, M., and Ritskes-Hoitinga, M., *Enhancing search efficiency by means of a search filter for finding all studies on animal experimentation in PubMed.* Lab Anim, 2010. **44**(3): p. 170-5.

3. de Vries, R.B., Hooijmans, C.R., Tillema, A., Leenaars, M., and Ritskes-Hoitinga, M., *Updated version of the Embase search filter for animal studies.* Lab Anim, 2014. **48**(1): p. 88.

4. Jüni, P., Holenstein, F., Sterne, J., Bartlett, C., and Egger, M., *Direction and impact of language bias in meta- analyses of controlled trials: empirical study.* International Journal of Epidemiology, 2002. **31**(1): p. 115-123.

5. Rohatgi, A. *WebPlotDigitizer* 2017; Available from: <http://arohatgi.info/WebPlotDigitizer>

6. Vesterinen, H.M., Sena, E.S., Egan, K.J., Hirst, T.C., Churolov, L., Currie, G.L., et al., *Meta-analysis of data from animal studies: a practical guide.* J Neurosci Methods, 2014. **221**: p. 92-102.

7. Harris, P.A., Taylor, R., Thielke, R., Payne, J., Gonzalez, N., and Conde, J.G., *Research electronic data capture (REDCap)—a metadata-driven methodology and workflow process for providing translational research informatics support.* Journal of biomedical informatics, 2009. **42**(2): p. 377-381.

8. Hooijmans, C.R., Rovers, M.M., de Vries, R.B., Leenaars, M., Ritskes-Hoitinga, M., and Langendam, M.W., *SYRCLE's risk of bias tool for animal studies.* BMC Med Res Methodol, 2014. **14**: p. 43.

9. Macleod, M.R., Lawson McLean, A., Kyriakopoulou, A., Serghiou, S., de Wilde, A., Sherratt, N., et al., *Risk of Bias in Reports of In Vivo Research: A Focus for Improvement.* PLoS Biol, 2015. **13**(10): p. e1002273.

10. Tipton, E., *Small sample adjustments for robust variance estimation with meta-regression.* Psychol Methods, 2015. **20**(3): p. 375-93.

**3 Figure Legends**

**Supplementary Table 1** Protocol Deviations Adapted from Moreau, D., & Gamble, B. (2020, January 7). Conducting a Meta-Analysis in the Age of Open Science: Tools, Tips, and Practical Recommendations. https://doi.org/10.31234/osf.io/t5dwg

**Supplementary Figure 1** Five levels of dependencies identified in the meta-analysis. The main mixed effect model is a multilevel model nesting the effect sizes within measurement type, study groups, and articles. Furthermore, the effect size weights are nested within control groups

**Supplementary Table 2** Proportion of stress types used across articles

**Supplementary Figure 2** (A) Funnel plot of all 165 effect sizes using nested weights (B) Funnel plot of the largest effect size for each of the 26 articles in the meta-analysis using nested weights

**Supplementary Table 3** Cannabinoid-Related Effects ↓ and ↑ represent a statistically significant decrease or increase (respectively) while ↔ represents a non-significant or unclear change. Drug doses are administered intraperitoneally and consistent within the article unless stated otherwise. LC-MRM – liquid chromatography multiple reaction monitoring, qRT-PCR - quantitative real time polymerase chain reaction, ChIP – chromatin immunoprecipitation, double *in situ* – double *in situ* hybridization, LC-MS – liquid chromatography mass spectrometry, FAAH – fatty acid amide hydrolase, CBD – cannabidiol, AEA – anandamide, PEA – palmitoyl-ethanolamide, OEA – oleoyl-ethanolamide, 2-AG – 2-arachydonylglycerol, MAGL – monoacylglycerol lipase, CB1/CB1R – cannabinoid 1 receptor, CB2R – cannabinoid 2 receptor H3K9ac – histone 3 lysine 9 acetylation H4K8ac – histone 4 lysine 8 acetylation, GAD65 – glutamic acid decarboxylase 65 kilodalton

**Supplementary Table 4** Stress-Related Effects ↓ and ↑ represent a statistically significant decrease or increase (respectively) while ↔ represents a non-significant or unclear change. Drug doses are administered intraperitoneally and consistent within the article unless stated otherwise. p.o. – oral, i.v. – intravenous, ELISA – enzyme-linked immunosorbent assay, qRT-PCR - quantitative real time polymerase chain reaction, IHC – immunohistochemistry, Electrophys – electrophysiology, rTMS – repetitive transcranial magnetic simulation, CBD – cannabidiol, BDNF – brain derived neurotropic factor, BrdU – bromodeoxyuridine, DCX – doublecortin, NeuN – neuronal nuclear protein, fEPSP – field excitatory post synaptic potential, LTP – long term potentiation
