# Supplementary figures and images for: The antidepressant and anxiolytic effects of cannabinoids in chronic unpredictable stress: a preclinical systematic review and meta-analysis
Source: Transl Psychiatry. 2022 May 31;12:217. doi: 10.1038/s41398-022-01967-1 (PMC9156762; doi:10.1038/s41398-022-01967-1)

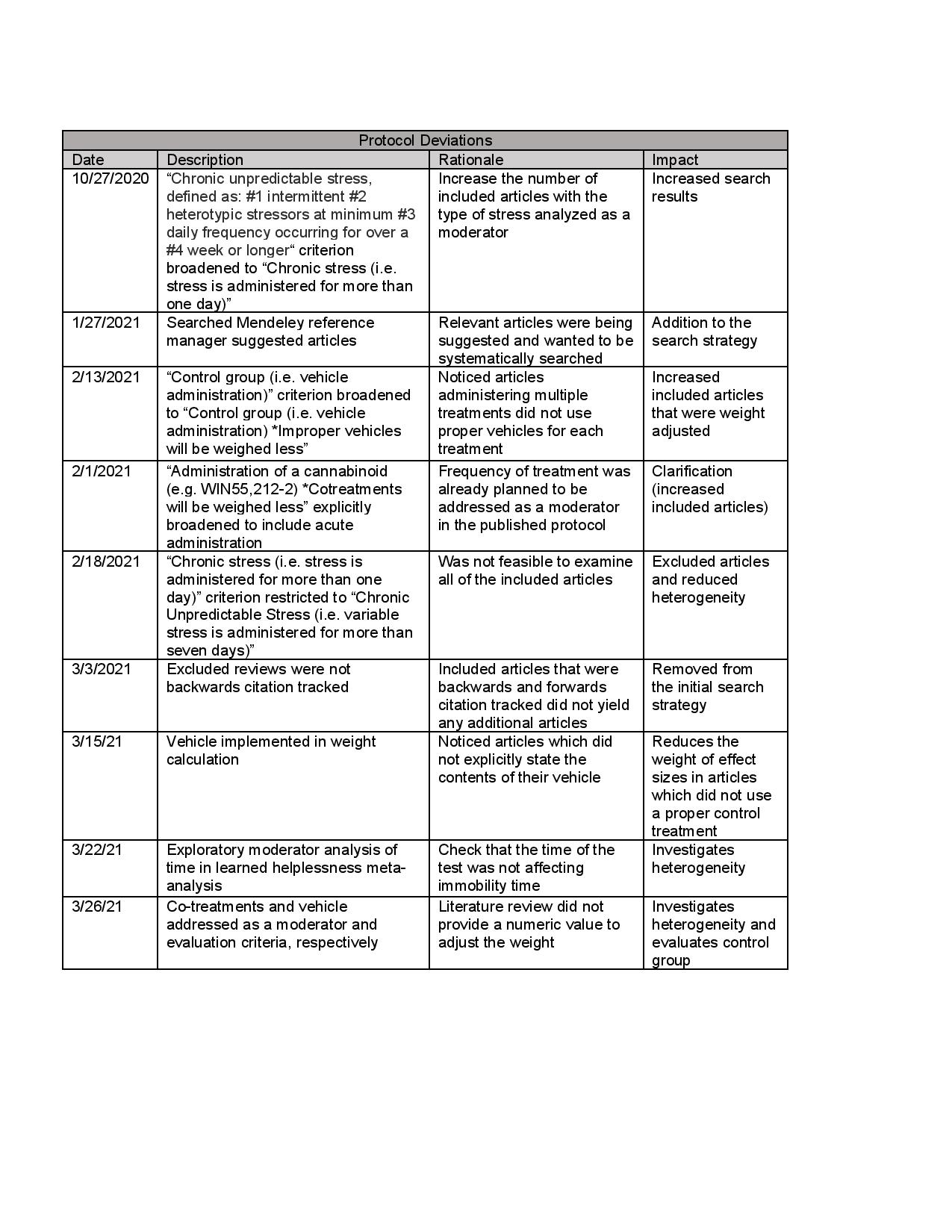

Supplement: Supplementary file 2 — Supplementary Table 1 [file 41398_2022_1967_MOESM2_ESM.jpg]

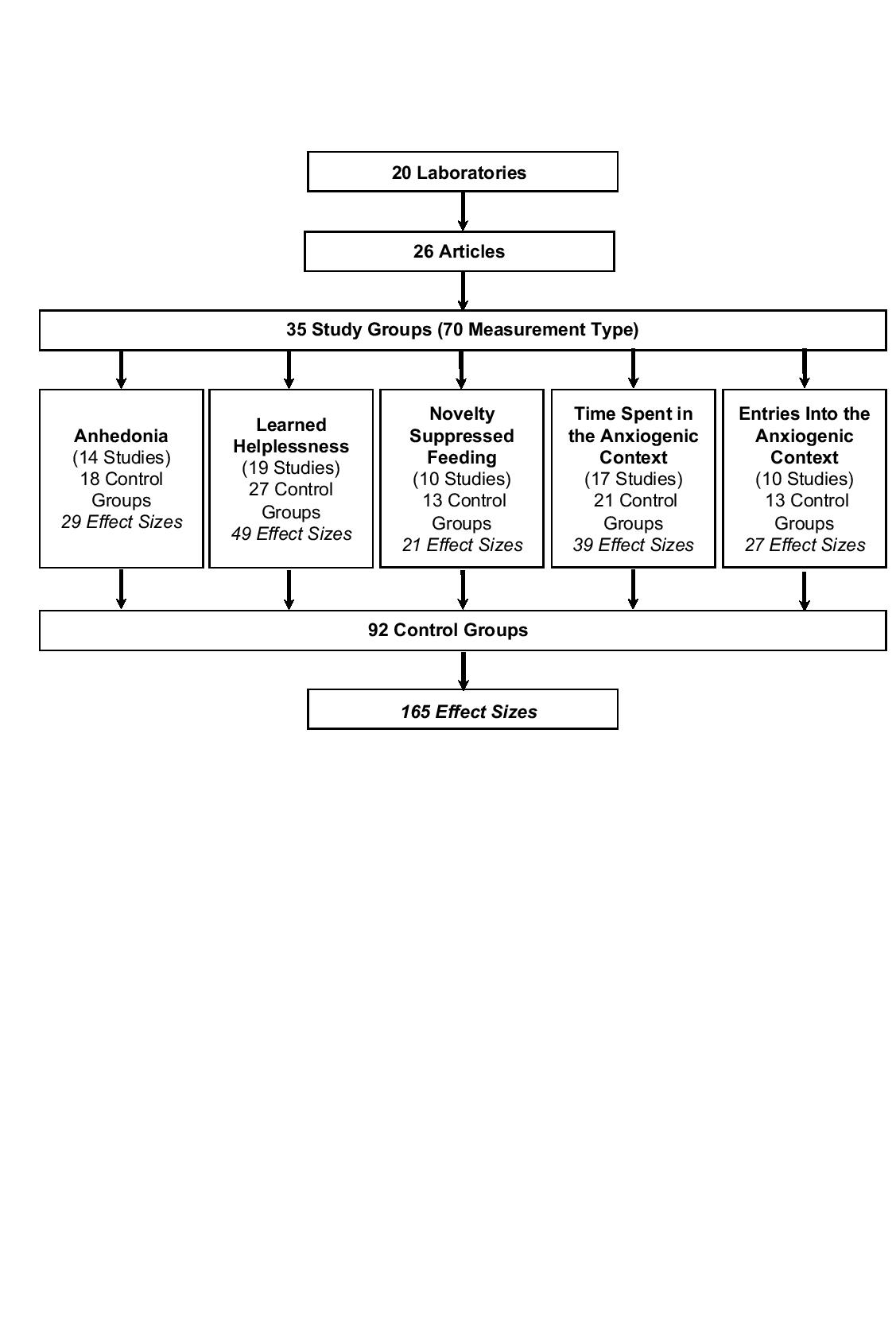

Supplement: Supplementary file 3 — Supplementary Figure 1 [file 41398_2022_1967_MOESM3_ESM.jpg]

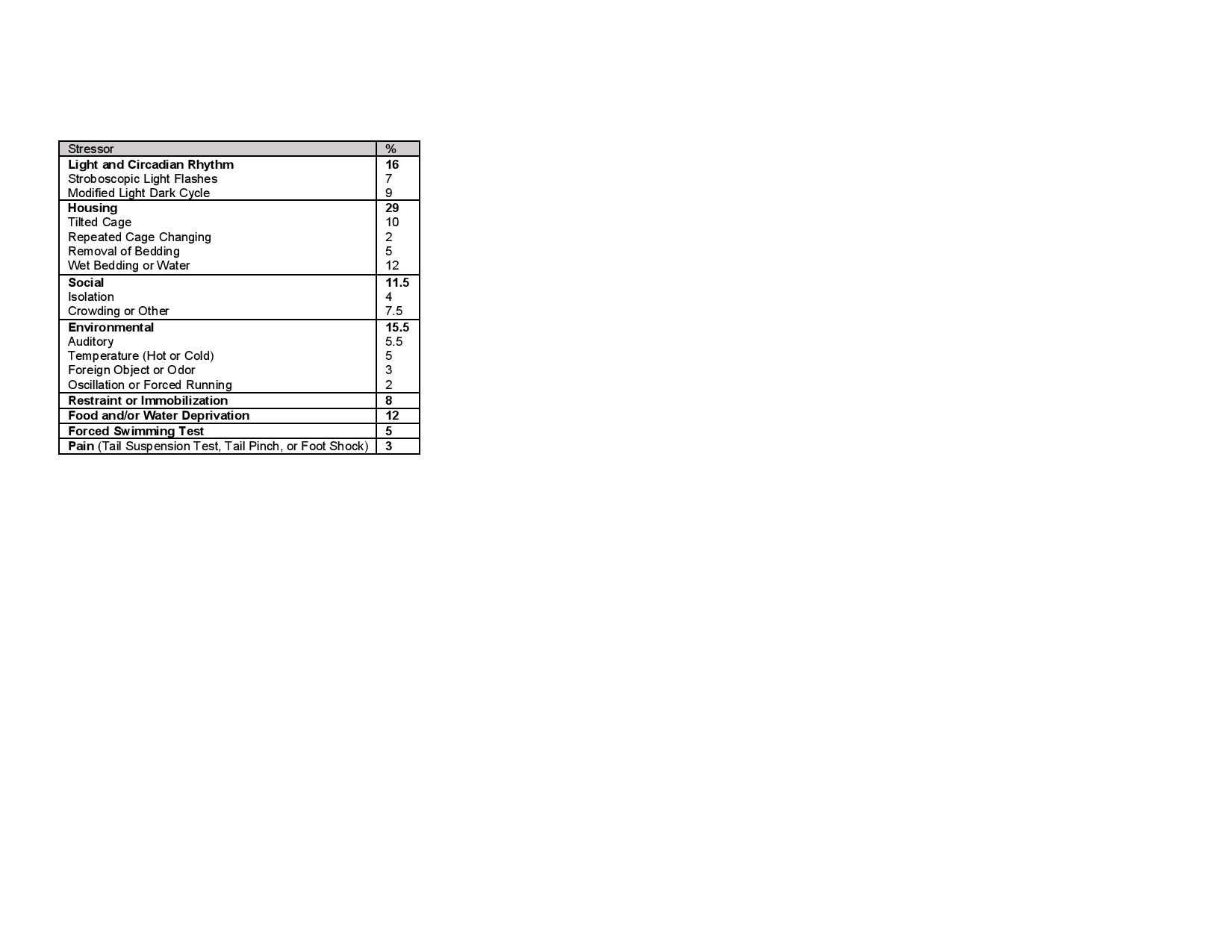

Supplement: Supplementary file 4 — Supplementary Table 2 [file 41398_2022_1967_MOESM4_ESM.jpg]

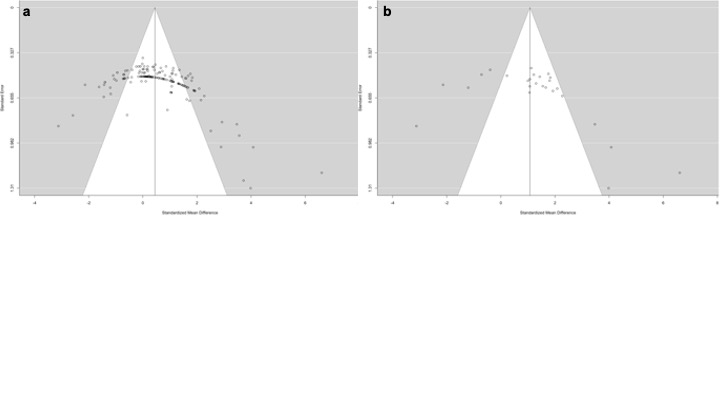

Supplement: Supplementary file 5 — Supplementary Figure 2 [file 41398_2022_1967_MOESM5_ESM.jpg]

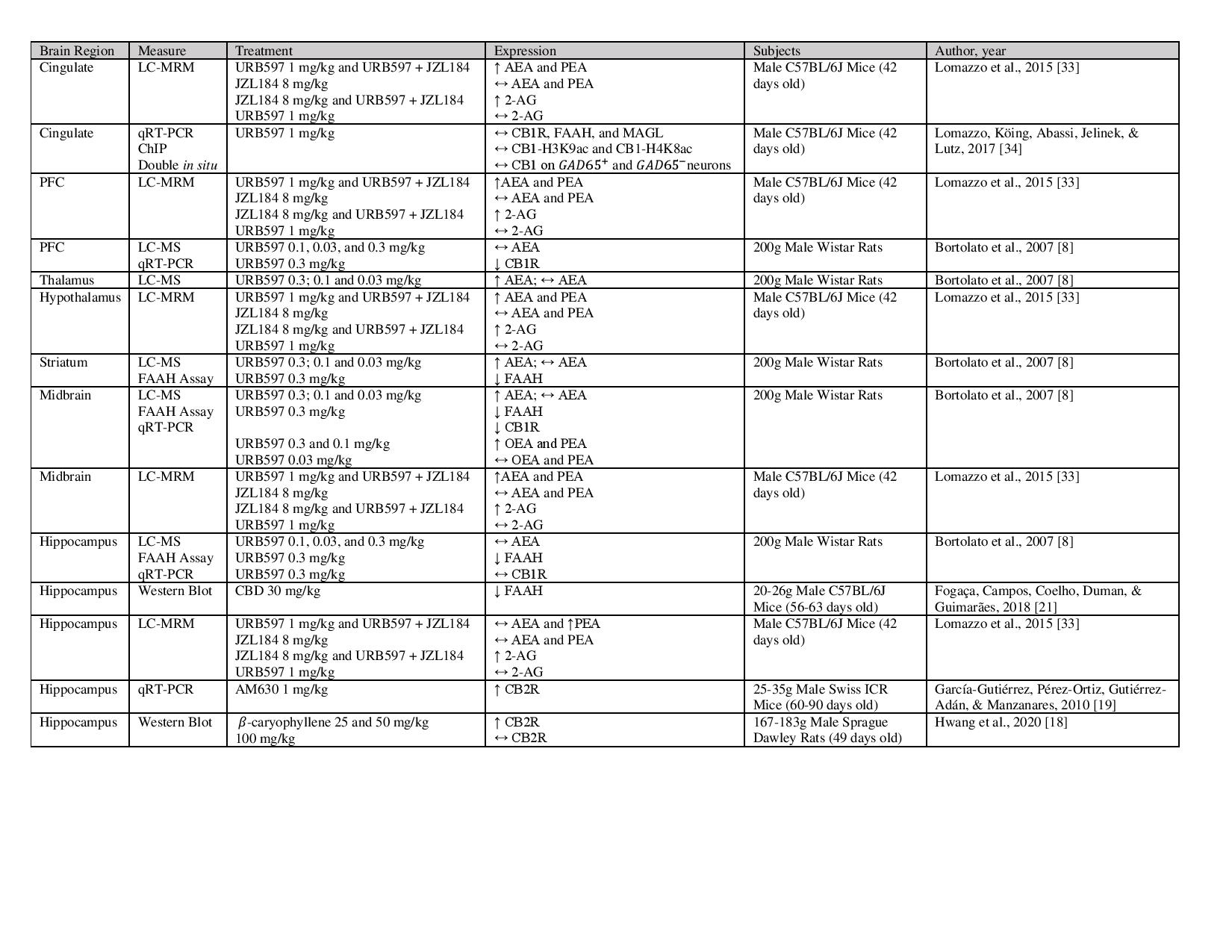

Supplement: Supplementary file 6 — Supplementary Table 3 [file 41398_2022_1967_MOESM6_ESM.jpg]

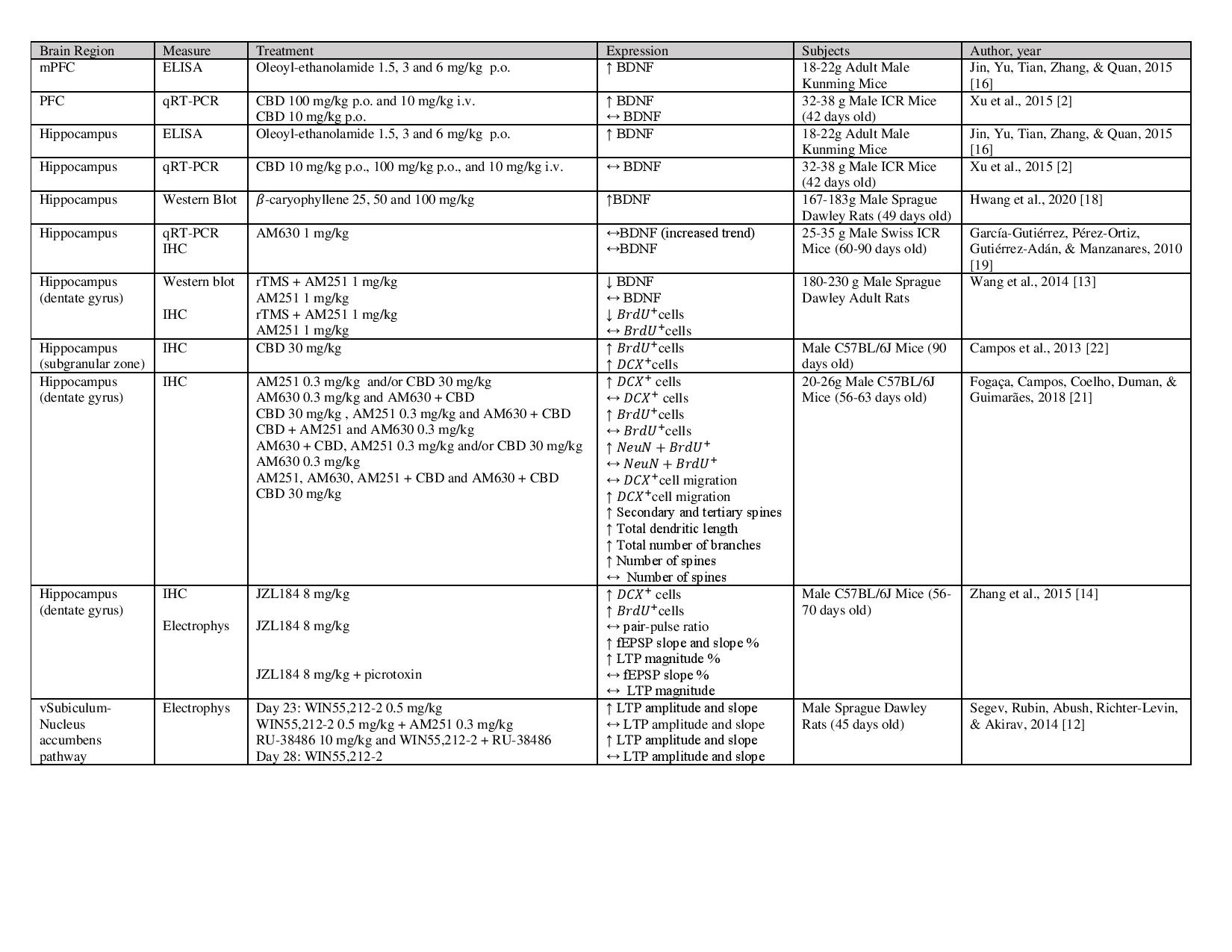

Supplement: Supplementary file 7 — Supplementary Table 4 [file 41398_2022_1967_MOESM7_ESM.jpg]
